# Antimicrobial resistant *Helicobacter fennelliae* isolated from non-diarrheal child stool sample in Battambang, Cambodia

**DOI:** 10.1186/s13099-018-0246-9

**Published:** 2018-05-30

**Authors:** Supaporn Ruksasiri, Woradee Lurchachaiwong, Patcharawalai Wassanarungroj, Oralak Serichantalergs, Chiek Sivhour, Nou Samon, Sovann Ly, Lon Chanthap, Ladaporn Bodhidatta, John Crawford

**Affiliations:** 1 Department of Enteric Diseases, Armed Forces Research Institute of Medical Sciences, 315/6 Rajvithi Road, Bangkok, 10400 Thailand; 2 Battambang Referral Hospital, PrekMohatep Village, SvayPor Commune, Battambang, Cambodia; 3 Armed Forces Research Institute of Medical Sciences, 18.118 Street Sangkat Mettapheap Khan 7 Makara, Phnom Penh, Cambodia; 4 Communicable Disease Control Department, Ministry of Health, 151-153, Kampuchea KromBlvd, Phnom Penh, Cambodia

**Keywords:** *Helicobacter fennelliae*, Non-diarrheal sample, Child, Cambodia, First-report

## Abstract

*Helicobacter fennelliae* (*H. fennelliae*) is associated with human gastroenteritis; however, *H. fennelliae* was isolated and confirmed by phenotypic and genotypic identification from a non-diarrheal child stool sample in Cambodia. Antimicrobial susceptibility testing demonstrated that this isolate had a high minimal inhibitory concentration against macrolides and quinolones, which are first-line antibiotic treatment choices for *Campylobacter* infections. Consequently, macrolides and quinolones were likewise expected to be ineffective against *Campylobacter*-like organisms such as *H. fennelliae*. This isolate warranted further genetic characterization to better understand associated antibiotic resistance mechanisms. Resistant pathogens from asymptomatic diarrheal cases are likely underestimated, and as such colonized individuals may spread resistant organisms to local community members and the environment.

## Background

*Helicobacter fennelliae* (*H. fennelliae*) is a new *Campylobacter* species originally isolated from asymptomatic, homosexual men with enteritis and proctitis in the past few decades [1]. Like *H. cinaedi*, this species is classified as enterohepatic *Helicobacter* that inhabits and causes bacteremia in intestinal and hepatobiliary tracts of various mammal and other species [2]. Additional evidence suggests that *H. fennelliae* was implicated as a contributing cause of human proctocolitis, gastroenteritis, and bacteremia, particularly in immunocompromised individuals [2, 3]. This *Helicobacter* species is a fastidious organism that is likely underestimated, and little is known about routes of transmission other than evidence indicates it is a zoonotic infection [2]. As a fastidious organism, molecular genotyping methods are recommended to identify *Helicobacter* species. Towards that end, the groEL and hsp60 genes encode a 60 kDa chaperonin protein present in virtually all eubacteria, some archaea, and in the plastids and mitochondria of eukaryotes. The utility of this target for bacterial species identification, detection, quantification, phylogenetic analysis, and microbial community profiling was well established [4]. Treatment recommendation guidelines are still not available for enterohepatic *Helicobacter* species. Various individual and combined antibiotic regimens were successfully used in treating *Helicobacter* infections; however, there is insufficient information to determine resistance rates of *H. fennelliae.* The main objective of this report is to describe phenotypic, genotypic, and antimicrobial susceptibility (AST) data from this *H. fennelliae* isolate from the stool of non-diarrheal child in Cambodia.

## Methods

A surveillance study to describe diarrhea etiologic agents in children and military personnel in Battambang, Cambodia has been conducted from 2014 until present. Both diarrheal and non-diarrheal stool samples were observed by microscopic examination for the presence of parasites, protozoa, and larvae. Samples were also assessed for the presence of *Giardia*, *Cryptosporidium* by enzyme-linked immunosorbent assay (ELISA), and for diarrheagenic *E. coli* by polymerase chain reaction (PCR) [5]. Enteric pathogens, including *Campylobacter* species, were isolated and identified by traditional culture methods [6]. The suspected *Campylobacter*-like colonies were subcultured on blood agar supplemented with 6% sodium formate and fumarate for 48–72 h at 37 °C under microaerobic conditions (10% CO_2_ and 5% O_2_). The biochemical identifications were included oxidase, catalase, indoxyl hydrolysis, hippurate hydrolysis, nitrate reduction, urease, hydrogen sulfide production, susceptibility to cephalothin and nalidixic acid (30 µg disc) (BD, Spark, USA), oxygen and temperature tolerance test. According to no antimicrobial susceptibility recommendation guidelines, *H. fennelliae* resistance was determined using the minimal inhibitory concentration (MIC) by E test (Liofilchem, Roseto degli Abruzzi TE, Italy) against azithromycin (AZM), erythromycin (ERY), nalidixic acid (NAL), ciprofloxacin (CIP), levofloxacin (LEV), ceftriaxone (CRO), spectinomycin (SPT), and tetracycline (TET). *C. jejuni* ATCC 33560 was used as a quality control strain.

Genomic DNA of suspected *Campylobacter*-like colonies was extracted and subsequently confirmed as belonging to the *Campylobacter* genus by screening for the 16S rRNA gene [7]. To determine *Campylobacter* species, the 15 primer sets of *cpn60* target gene were used for verified species as described elsewhere [7, 8]. Subsequently, the unknown *Campylobacter* species beyond 15 primer sets identification were further sequencing analysis by amplifying *cpn60* target gene with degenerate primers H729 and H730 [4]. The sequences of degenerate primers were H729: 5′-CGCCAGGGTTTTCCCAGTCACGACGAIIIIGCIGGIGAYGGIACIACIAC-3′ and H730 5′-AGCGGATAACAATTTCACACAGGAYKIYKITCICCRAAI CCIGGIGCYTT-3′. PCR amplification was carried out in a total volume of 50 µL containing 6 µL of genomic DNA template, 2.5 U AmpliTaq Gold^®^ DNA polymerase (Applied Biosystems, Foster City, Calif.), 5 mM MgCl_2_, 100 µM each of the dNTPs and 50 nM each of degenerate primers [4]. The cycling conditions were performed at 94 °C for 5 min, followed by 28 cycles of 1 min at 94 °C, 1 min at 46 °C, 1 min at 72 °C, and a final extension at 72 °C for 10 min. The purified PCR products were additionally differentiate *Campylobacter* species from *Helicobacter* and *Acrobacter* species using primers M13F-pUC (− 40) 5′-GTTTTCCCAGTCACGAC-3′ and M13R (− 20) 5′-GCGGA-TAACAATTTCACACAGG-3′. The result of partial *cpn60* sequences (555 bp) was compared with the database in cpnDB (http://cpndb.cbr.nrc.ca) [4]. The confirmed partial sequence was submitted to the National Center for Biotechnology Information (NCBI) before constructing phylogenetic analysis by BioNumerics software version 7.6 (Applied Maths, Belgium).

## Results and discussion

A non-diarrheal stool sample of a young child who presented to the hospital with fever and, cough was submitted for laboratory testing. The stool characteristic was loose without mucus, blood, RBCs, or WBCs. No gastrointestinal parasites were detected microscopically or by ELISA. Other enteric bacterial pathogens, including diarrheagenic *E. coli*, were not identified, except for suspected colonies of a *Campylobacter*-like organism. The colonies characteristics which were presented after 6 days incubation were thin, flat, film-like colony, with a hypochlorite odor. Biochemical reactions of the colony were positive for oxidase, catalase, and indoxyl acetate hydrolysis. It was susceptible to cephalothin disk but resistant to nalidixic acid disk and could be grown at 42 °C under microaerobic conditions. Culture results indicated that *H. fennelliae* grows well by supplementing 6% sodium formate and fumarate in blood agar. This is likely due to the fact that formate replaces hydrogen as the electron donor, and fumarate serves as the terminal electron acceptor for hydrogen-required organism growth [9]. Notably, an absence of hydrogen, the low-cost supplemented media, and a long incubation period are suggested to support growth of *H. fennelliae*.

The MIC results of *H. fennelliae* and *C. jejuni* ATCC 33560 were presented in Table 1. Results for this *H. fennelliae* isolate demonstrated high MICs to macrolides and quinolones, consistent with previous studies [10, 11] and similar to *H. cinaedi* data [12]. Macrolides, generally considered the drug of choice for *Campylobacter* treatment [1], may be clinically less effective for *Campylobacter*-like organism infections such as *H. fennelliae* and *H. cinaedi*. Little is known about the antimicrobial resistance mechanisms of *H. fennelliae*. Mutations of the gyrase and 23S rRNA genes may be responsible for decreased susceptibility to quinolones and macrolides, respectively [10]. However, decreased susceptibility to low MIC macrolide levels were mentioned in a previous study [13]. The *H. fennelliae* isolate from our study exhibited a high MIC to macrolides, warranting further molecular characterization to explore other resistance mechanisms.

**Table 1:** Determination the minimal inhibitory concentration (MIC) results of *H. fennelliae* and *C. jejuni* ATCC 33560 against azithromycin (AZM), erythromycin (ERY), nalidixic acid (NAL), ciprofloxacin (CIP), levofloxacin (LEV), tetracyclin (TET), ceftriaxone (CRO) and spectinomycin (SPT)

Isolates	MIC (µg/mL)							
	AZM	ERY	NAL	CIP	LEV	TET	CRO	SPT
*H. fennelliae*	≥ 256	≥ 256	≥ 256	≥ 32	3	0.125	0.125	4
*C. jejuni* ATCC33560	0.125	0.75	4	0.094	0.25	0.25	≥ 32	0.5

**Fig. 1:**
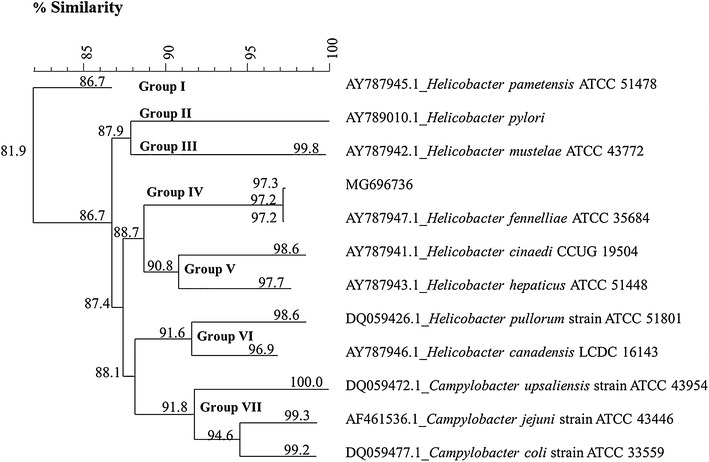
Neighbor-joining phylogenetic trees based on partial *cpn60* gene sequences (555 bp) of *Helicobacter* strain (MG696736), as compared to *cpn60* sequences of other *Helicobacter*, and *Campylobacter* strains in cpnDB database

The genotyping confirmation of this *H. fennelliae* isolate was performed by sequencing the *cpn60* gene, and the result was submitted to NCBI under the accession number MG696736. A phylogenetic tree analysis (Fig. 1) divided *Helicobacter* and *Campylobacter* strains into seven distinct groups (cut-off of 90%). The MG696736 entry was classified as group IV, which was 97.2% similar to *H. fennelliae* ATCC 35684, whereas *Campylobacter* species was classified as group VII, which is distinct from the *Helicobacter* group VI (cut-off of 90%).

*H. fennelliae* was suggested as a significant pathogen associated with human gastroenteritis; however, its prevalence and antimicrobial resistant profile might be considerably underestimated due to inadequate isolation and identification methods [14]. To the best of our knowledge, this is the first report of a macrolide and quinolone resistant *H. fennelliae* identified in a young Cambodian child asymptomatic for intestinal infection. This isolate resembles *H. fennelliae*, which was previously identified in a boy suffering gastroenteritis and is also isolated from dog specimens [15]. With the introduction of the ‘Cape Town Protocol,’ *H. fennelliae* may be isolated from stool and blood culture in an H_2_-rich microaerophilic atmosphere. Prior evidence indicated that *Helicobacter* species related to *H. fennelliae* were isolated from blood of a young child suffering diarrhea symptoms [16]. Nevertheless, the nucleotide sequences of *H. fennelliae* obtained from blood and stool were not significantly different [17]. Unfortunately blood samples were not available from the child in this study, so that comparison was not achievable. *H. fennelliae* was predominantly isolated from children who presented with diarrheal symptoms, although stools from asymptomatic diarrheal children with asthma and/or failure to thrive (FTT) were also positive for *H. fennelliae* [16, 17]. Another possible explanation of this *H. fennelliae* finding in stool of asymptomatic diarrheal Cambodia child could relate to breastfeeding. Evidence suggests that maternal milk contains a variety of functionally bioactive agents from her innate immune system [18], as well as a mechanism to influence microbial changes in the infant’s gastrointestinal system [19]. As a result of widespread breastfeeding campaigns in the developing world, this may play an important role in the level of asymptomatic carriage within a community [18, 20]. The association between asymptomatic carriage and diarrheal pathogens such as *Salmonella*, *E. coli* O157 and *Campylobacter* was previously reported in outbreaks elsewhere [20]. Identification of an antibiotic resistant *H. fennelliae* strain from an asymptomatic diarrheal person would probably be transmitted into local communities and environmental contamination. Hence, the public health significance of resistant pathogens in human feces warrants effective monitoring to prevent disease outbreaks.

In conclusion, phenotypic and genotypic assessments confirmed that *H. fennelliae* was isolated from a non-diarrheal stool sample of a Cambodian child suffering from fever with cough and convulsion. The supplement media, incubation atmosphere, and incubation period utilized permitted culture, isolation, and identification of *H. fennelliae.* The high MICs values against macrolides (AZM, ERY) and quinolones (NAL, CIP) indicated these are less effective against *H. fennelliae*. This isolate should be further characterized to better understand associated resistance mechanisms.

## References

[1] Totten PA, Fennell CL, Tenover FC, Wezenberg JM, Perine PL, Satamm WE (1985). *Campylobacter cinaedi* (sp.nov.) and *Campylobacter fennelliae* (sp. Nov): two new *Campylobacter* species associated with enteric diseases in homosexual men. J Infect Dis.

[2] James HJ, Michael AP, Karen CC, Guido FMLL, Sandry SR, David WW (2015). Manual of clinical microbiology.

[3] O’Rourke JL, Grehan M, Lee A (2001). Non-pylori *Helicobacter* species in humans. Gut.

[4] Hill JE, Paccagnellla A, Law K, Melito PL, Woodward DL, Price L (2006). Identification of *Campylobacter* spp. and discrimination from *Helicobacter* and *Arcobacter* spp. by direct sequencing of PCR-amplified *cpn60* sequences and comparison to cpnDB, a chaperonin reference sequence database. J Med Microbiol.

[5] Meng CY, Smith BL, Bodhidatta L, Richard SA, Vansith K, Thy B (2011). Etiology of diarrhea in young children and patterns of antibiotic resistance in Cambodia. Pediatr Infect Dis J.

[6] Garcia LS. Clinical microbiology procedures handbook. 3rd ed, Washington, D.C.: ASM Press; 2010. P. 3.8.1.1–3.8.2.16 and 3.17.1.1–3.18.2.1.

[7] Bullman S, O’Leary J, Corcoran D, Sleator RD, Lucey B (2012). Molecular-based detection of non-culturable and emerging campylobacteria in patients presenting with gastroenteritis. Epidemiol Infect.

[8] Chaban B, Musil KM, Himsworht CG, Hill JE (2009). Development of cpn60-based Real-time quantitative PCR assays for the detection of 14 *Campylobacter* species and application to screening of canine fecal samples. Appl Environ Microbiol.

[9] Roop RM, Smibert RM, Johnson JL, Krieg NR (1985). *Campylobacter mucosalis* (Lawson, Leaver, Pettigrew, and Rowland 1981) comb. nov.: emended description. Int J Syst Bacteriol.

[10] Rimbara E, Mori S, Kim H, Matsui M, Suzuki S, Takahashi S (2013). *Helicobacter cinaedi* and *Helicobacter fennelliae* transmission in a hospital from 2008 to 2012. J Clin Microbiol.

[11] Fujiya Y, Nagamatsu M, Tomida J, Kawamura Y, Yamamoto K, Mawatari M (2016). Successful treatment of recurrent *Helicobacter fennelliae* bacteraemia by selective digestive decontamination with kanamycin in a lung cancer patient receiving chemotherapy. JMM Case Rep.

[12] Kawamura Y, Tomida J, Morita Y, Fujii S, Okamoto T, Akaike T (2014). Clinical and bacteriological characteristics of *Helicobacter cinaedi* infection. J Infect Chemother.

[13] Hsueh PR, Teng LJ, Hung CC, Chen YC, Yang PC, Ho SW (1999). Septic shock due to *Helicobacter fennelliae* in a non-human immunodeficiency virus-infected heterosexual patient. J Clin Microbiol.

[14] Lastovica AJ (2006). Emerging *Campylobacter* spp.: the tip of the iceberg. Clin Microbiol Newsl.

[15] Burnens AP, Stanley J, Schaad UB, Nicolet J (1993). Novel Campylobacter-like organism resembling *Helicobacter fennelliae* isolated from a boy with gastroenteritis and from dogs. J Clin Microbiol.

[16] Tee W, Hinds S, Montgomery J, Dyall-Smith ML (2000). A probable new *Helicobacter* species isolated from a patient with bacteremia. J Clin Microbiol.

[17] Smuts HE, Lastovica AJ (2011). Molecular characterization of the 16S rRNA Gene of *Helicobacter fennelliae* isolated from stools and blood cultures from paediatric patients in South Africa. J Pathog.

[18] Morrow AL, Ruiz-Palacios GM, Altaye M, Jiang X, Guerrero ML, Meinzen-Derr JK (2004). Human milk oligosaccharides are associated with protection against diarrhea in breast-fed infants. J Pediatr.

[19] Ogbo FA, Agho K, Ogeleka P, Woolfenden S, Page A, Eastwood J, Global Child Health Research Interest Group (2017). Infant feeding practices and diarrhoea in sub-Saharan African countries with high diarrhoea mortality. PLoS ONE.

[20] Quilliam RS, Cross P, Williams AP, Edwards-Jones G, Salmon RL, Rigby D (2013). Subclinical infection and asymptomatic carriage of gastrointestinal zoonoses: occupational exposure, environmental pathways, and the anonymous spread of disease. Epidemiol Infect.

